# Trends and Patterns in Emergency Department Visits: A Comprehensive Analysis of Adult Data From the National Center for Health Statistics (NCHS) Database

**DOI:** 10.7759/cureus.66059

**Published:** 2024-08-03

**Authors:** Mohamed M Ohaiba, Eberechukwu G Anamazobi, Okelue E Okobi, Kayode Aguda, Victor U Chukwu

**Affiliations:** 1 Industrial Engineering, Louisiana State University, Baton Rouge, USA; 2 Surgery, American International School of Medicine, Georgetown, GUY; 3 Internal Medicine, South Atlanta Primary Care, Atlanta, USA; 4 Family Medicine, Larkin Community Hospital Palm Springs Campus, Miami, USA; 5 Family Medicine, Medficient Health Systems, Laurel, USA; 6 Family Medicine, Lakeside Medical Center, Belle Glade, USA; 7 Emergency Medicine, College of Health Sciences, Obafemi Awolowo University, Ile-Ife, NGA; 8 Medicine, Abia State University, Uturu, NGA

**Keywords:** retrospective data analysis, trends, adults, nchs, emergency department visits

## Abstract

Background

Emergency department (ED) visits among adults have increased in recent years, with the United States reporting 140 million ED visits in 2021, equating to an overall rate of 43 visits per 100 people. This trend underscores challenges in accessing primary care and addressing underlying health conditions. Understanding the trends and patterns in ED utilization is essential for informing healthcare policy and practice.

Objective

This study aims to comprehensively analyze trends and patterns in ED visits among adults using data from the National Center for Health Statistics (NCHS) database.

Methods

We conducted a retrospective analysis of ED visit data from 1999 to 2019, focusing on adults aged 18 and over. The prevalence rates of ED visits were examined across demographic, socioeconomic, and geographic groups using datasets retrieved from the NCHS database. Statistical analysis included one-way ANOVA and chi-square tests to assess variations in ED visit rates.

Results

This study’s findings revealed a consistent increase in overall ED visits among adults, from 17.2 ± 0.3% in 1999 to 21.7 ± 0.3% in 2019. Disparities in ED utilization were evident across demographic and socioeconomic groups. Females had slightly higher visit rates, and significant racial disparities were noted, with American Indian or Alaska Native and Black or African American individuals showing the highest visit rates. Age-specific variations were observed, with young adults (18-24 years) and older adults (65 years and above) exhibiting higher visit rates. Socioeconomic status and health insurance coverage emerged as significant determinants, highlighting disparities in healthcare access.

Conclusion

This study provides valuable insights into the trends and patterns of ED visits among adults, emphasizing the need for targeted interventions to address healthcare disparities and improve access to primary care services.

## Introduction

Emergency department (ED) visits are vital components of healthcare delivery, serving as the primary site for acute medical care, particularly for individuals with urgent medical needs. The frequency and nature of ED utilization offer critical insights into the healthcare-seeking behaviors of the population, reflecting both individual health concerns and systemic healthcare dynamics [1,2]. The healthcare landscape in the United States has undergone significant transformations over the past few decades, marked by shifts in insurance coverage, advancements in medical technology, and changes in healthcare policies [3]. These transformations have influenced how individuals access and utilize healthcare services, including emergency care. Therefore, examining ED utilization patterns within a specified timeframe, such as the past 12 months, provides a snapshot of current trends and effectively informs strategies for addressing healthcare needs [3,4].

A total of 140 million ED visits were reported in the United States in 2021, with an overall rate of 43 visits per 100 people [5]. Adults aged 75 and over had 66 visits per 100, while 18% of adults visited the ED in the past year, highlighting diverse healthcare needs [5]. The demographic diversity of individuals seeking care in EDs reflects varied socioeconomic, racial, and geographic backgrounds. Disparities in healthcare access contribute to differing ED utilization rates among demographic groups. Analyzing ED visits among adults aged 18 and over helps identify vulnerable populations, assess care barriers, and enhance healthcare equity [6]. Understanding the reasons for ED visits is vital for resource allocation and healthcare delivery improvement. Some visits stem from emergencies, while others result from limited primary care access, health literacy issues, or preventive service gaps. Identifying the primary reasons for ED use enables targeted interventions to reduce unnecessary visits and promote preventive and continuous care [7,8].

The National Center for Health Statistics (NCHS) provides essential health-related data, aiding research with extensive demographics and health indicators. Researchers leverage the rich dataset the NCHS survey provides to address a wide range of research questions and policy concerns. From assessing disparities in healthcare access and utilization to evaluating the effectiveness of public health interventions, the NCHS database survey informs evidence-based decision-making at both the national and local levels [9].

This study examines ED visits among adults by leveraging NCHS data from annual household surveys spanning 1999-2019. By analyzing the dataset, the research aims to understand ED, demographic disparities, and socioeconomic patterns and optimize emergency healthcare delivery. The research identifies high-risk populations through temporal trend and subgroup analyses and informs evidence-based policies and interventions for diverse United States healthcare needs.

## Materials and methods

Data source and study design

A retrospective observational analysis was conducted using data from the NCHS database, covering a substantial period from 1999 to 2019. Acknowledged as a pivotal repository of health-related information in the United States, the NCHS database served as the primary data source for this investigation, offering a comprehensive collection of demographic, health, and geographical data obtained through various surveys, including the National Health Interview Survey (NHIS).

Study participants and questionnaire

The inclusion criteria comprised adults aged 18 and above who participated in relevant surveys conducted by the NCHS during the study period. In the primary NHIS survey, respondents were asked, “During the past 12 months, how many times have you gone to a hospital emergency room about your own health? This includes emergency room visits that resulted in hospital admission.”

Data collection and quality assurance

Datasets were systematically retrieved from the NCHS database using standardized data extraction protocols. Rigorous attention was given to data quality and consistency, ensuring precision in the analysis. Data cleaning procedures involved addressing missing values, outliers, and inconsistencies. The dataset was strategically aggregated by year, age group, gender, and other pertinent categories to facilitate a thorough and structured statistical examination.

Variables of interest

The primary focus of the study was on ED visits within the past 12 months among adults aged 18 and over. Demographic data included age (categorized into six groups), gender (male/female), and race/ethnicity (Hispanic/Latino, Black or African American, White, Asian). Socioeconomic factors, such as poverty level (four categories: below 100%, 100-199%, 200-399%, and 400% or more), insurance status, and location of residence, such as metropolitan statistical areas (MSAs), were also analyzed.

Data analysis and statistical methods

The NCHS datasets underwent rigorous cleaning and preprocessing to ensure data integrity, including addressing missing values, outliers, and inconsistencies. Statistical analysis utilized a one-way ANOVA (for age group, racial category, poverty level, and health insurance status) and chi-square t-test (for gender) to examine variations in ED visit rates across different demographic attributes and socioeconomic indicators. A significance level of <0.05 was employed to determine statistical significance. The analyses were conducted using Microsoft Excel (Microsoft Corporation, Redmond, United States).

Ethical considerations

This study utilized de-identified, publicly available data from the NCHS, ensuring participant privacy and confidentiality. No additional ethical approval was required for the analysis of the secondary data collected. All data handling procedures strictly adhered to the confidentiality standards outlined by the NCHS, maintaining the utmost privacy of participants. These rigorous ethical considerations enhance the credibility and acceptability of the study for publication in scientific journals.

## Results

The analysis of ED visits within the past 12 months among adults revealed notable trends over the 21-year period from 1999 to 2019. The prevalence of ED visits ranged from 17.2 ± 0.3% (1999) to 21.7 ± 0.3% (2019) during the study period. Notable changes were observed, with the lowest prevalence recorded in 2015 (18.6 ± 0.3%) and the highest in 2019 (21.7 ± 0.3%).

Based on gender

When stratified by gender, males demonstrated a prevalence ranging from 16.1 ± 0.4% to 19.4 ± 0.5% between 1999 and 2019. Notably, the lowest prevalence was observed in 2014 (16.5 ± 0.4%), while the highest was in 2009 (19.3 ± 0.5%). Conversely, females displayed a higher prevalence, ranging from 18.2 ± 0.4% to 24 ± 0.4% during the same period. The lowest prevalence was recorded in 1999 (18.2 ± 0.4%), while the highest was in 2010 (24.3 ± 0.5%) (Table 1). Both sexes demonstrated significant differences in ED visit prevalence across the years (P < 0.05), suggesting distinct patterns in ED utilization between males and females.

**Table 1:** Study characteristics of ED visits within the past 12 months ED, emergency department; MSA, metropolitan statistical area

Characteristic	1999	2000	2001	2002	2003	2004	2005	2006	2007	2008	2009	2010	2011	2012	2013	2014	2015	2016	2017	2018	2019	P-value
Total, 18 years and over, age-adjusted	17.2 ± 0.3	20.2 ± 0.3	19.7 ± 0.3	20.5 ± 0.3	20 ± 0.3	20.7 ± 0.3	20.5 ± 0.3	20.5 ± 0.3	20.2 ± 0.3	20.7 ± 0.4	21.4 ± 0.3	21.4 ± 0.3	20.4 ± 0.3	19.5 ± 0.3	18.8 ± 0.3	18.6 ± 0.3	18.8 ± 0.3	19.4 ± 0.3	18.6 ± 0.3	21.3 ± 0.3	21.7 ± 0.3	-
Sex (one or more ED visits percentage (SE))																						
Male	16.1 ± 0.4	18.7 ± 0.4	18.8 ± 0.4	19.6 ± 0.4	18.2 ± 0.4	19.3 ± 0.4	18.6 ± 0.4	19 ± 0.5	18.4 ± 0.5	19.3 ± 0.5	19.9 ± 0.5	18.5 ± 0.4	18 ± 0.4	17 ± 0.4	16.5 ± 0.4	16.9 ± 0.4	16.9 ± 0.4	17 ± 0.4	16.3 ± 0.4	18.9 ± 0.5	19.4 ± 0.5	P < 0.05
Female	18.2 ± 0.4	21.6 ± 0.4	20.5 ± 0.4	21.5 ± 0.4	21.8 ± 0.4	22.1 ± 0.4	22.3 ± 0.4	22.1 ± 0.4	21.9 ± 0.4	22.1 ± 0.5	22.9 ± 0.5	24.3 ± 0.5	22.7 ± 0.4	22 ± 0.4	21 ± 0.4	20.3 ± 0.4	20.6 ± 0.4	21.7 ± 0.5	20.9 ± 0.5	23.6 ± 0.5	24 ± 0.4	
Age group (one or more ED visits percentage (SE))																						
18–24 years	21.7 ± 0.9	25.7 ± 0.9	23.8 ± 0.9	24.7 ± 0.9	23.9 ± 0.9	24.4 ± 1	25.3 ± 1	24.9 ± 1.1	23.3 ± 1.1	24.1 ± 1.1	24.6 ± 1.1	25.4 ± 1	23.8 ± 1	22.3 ± 0.9	20.8 ± 0.9	20.9 ± 1.1	20.5 ± 1.1	20.6 ± 1	19.2 ± 1.1	21 ± 1.1	21.2 ± 1.1	P < 0.05
25–44 years	16.5 ± 0.4	18.8 ± 0.4	18.3 ± 0.4	19.4 ± 0.4	18.6 ± 0.4	19.7 ± 0.4	19.2 ± 0.4	18.9 ± 0.5	19.3 ± 0.5	20.6 ± 0.6	21.1 ± 0.6	20.7 ± 0.5	19.5 ± 0.5	18.4 ± 0.5	17.7 ± 0.4	17.5 ± 0.5	17.9 ± 0.5	18.1 ± 0.5	17.4 ± 0.5	20.5 ± 0.6	21 ± 0.5	
45–54 years	14.3 ± 0.6	17.9 ± 0.6	17.7 ± 0.6	18.1 ± 0.6	17.8 ± 0.6	17.9 ± 0.6	17.6 ± 0.6	17.9 ± 0.7	18 ± 0.7	17.1 ± 0.7	18 ± 0.7	18.6 ± 0.7	18 ± 0.6	18 ± 0.6	17.2 ± 0.6	16.2 ± 0.6	16.4 ± 0.6	18.4 ± 0.7	17.1 ± 0.7	18.7 ± 0.8	20.1 ± 0.7	
55–64 years	15.1 ± 0.6	17 ± 0.7	18.5 ± 0.7	18.4 ± 0.7	19.3 ± 0.7	18.4 ± 0.7	19 ± 0.7	18.9 ± 0.8	18.7 ± 0.8	18.2 ± 0.8	18.9 ± 0.7	19.8 ± 0.7	18.5 ± 0.7	18 ± 0.7	18.1 ± 0.7	18.9 ± 0.7	18.4 ± 0.7	17.8 ± 0.7	18.4 ± 0.7	19 ± 0.7	20.3 ± 0.6	
65–74 years	17.3 ± 0.8	21.6 ± 0.8	19.7 ± 0.8	21.2 ± 0.8	19.7 ± 0.8	20.8 ± 0.9	20.8 ± 0.8	20.6 ± 1	20.2 ± 1	20.7 ± 1	21.6 ± 1	20.7 ± 0.9	20.4 ± 0.8	19.9 ± 0.8	18.4 ± 0.8	18.9 ± 0.8	18.3 ± 0.7	20.5 ± 0.8	18.5 ± 0.7	23 ± 0.8	22.6 ± 0.7	
75 years and over	23.1 ± 0.9	26.2 ± 0.9	25.4 ± 0.9	27.1 ± 1	26.6 ± 1	28.7 ± 1	27.1 ± 0.9	28.9 ± 1.3	26.5 ± 1.1	26.4 ± 1.2	28.8 ± 1.2	27.4 ± 1.1	27 ± 0.9	25.3 ± 1	25.3 ± 1	24.4 ± 0.9	26.7 ± 1	27.4 ± 1	27.6 ± 1	32.5 ± 1.1	30.9 ± 0.9	
Race (one or more ED visits percentage (SE))																						
White only	16.6 ± 0.3	19.4 ± 0.3	19 ± 0.3	19.6 ± 0.3	19.2 ± 0.3	20 ± 0.3	19.8 ± 0.3	20.1 ± 0.4	19.6 ± 0.4	20.4 ± 0.4	20.4 ± 0.4	20.7 ± 0.4	19.8 ± 0.3	18.8 ± 0.3	18.1 ± 0.3	17.7 ± 0.3	18 ± 0.4	18.9 ± 0.4	17.9 ± 0.4	20.6 ± 0.4	20.6 ± 0.4	P < 0.05
Black or African American only	22.2 ± 0.8	26.5 ± 0.8	25.2 ± 0.8	27.8 ± 0.8	27.8 ± 0.8	27 ± 0.9	26.3 ± 0.8	25.6 ± 0.8	26.3 ± 0.9	25.1 ± 0.9	31.1 ± 0.9	28.6 ± 0.9	28 ± 0.8	26.4 ± 0.8	25.4 ± 0.8	26.3 ± 0.8	26.6 ± 0.9	25.6 ± 0.9	25.3 ± 1.1	26.9 ± 1	29.3 ± 1	
American Indian or Alaska Native only	29.2 ± 4	30.3 ± 3.4	33.8 ± 4.7	25.3 ± 4.1	22.5 ± 3.7	27.8 ± 3.8	31 ± 4.6	21.1 ± 3.7	26.7 ± 3.3	30.2 ± 3	23.5 ± 3.1	22.6 ± 3	27.3 ± 3.1	22.1 ± 3	26.7 ± 3.1	31.1 ± 3.4	28.3 ± 3.3	26.7 ± 2.9	27.2 ± 2.9	31.7 ± 3	33.6 ± 3.7	
Asian only	9.7 ± 1.2	13.6 ± 1.3	12.7 ± 1.4	13.6 ± 1.4	12.9 ± 1.4	12.2 ± 1.2	15.4 ± 1.4	13.6 ± 1.1	11.9 ± 1.1	11.5 ± 1.1	13.2 ± 1.1	13.3 ± 1	9.9 ± 0.7	10.9 ± 0.9	10.1 ± 0.8	11 ± 1	9.4 ± 0.8	11.5 ± 1.1	11.7 ± 1	14.7 ± 1.3	13.2 ± 1.1	
Percent of poverty level (one or more ED visits percentage (SE))																						
Below 100%	26 ± 0.9	29 ± 0.9	26.5 ± 0.9	28.6 ± 0.9	26.3 ± 0.8	29.3 ± 0.9	29.8 ± 0.9	28.2 ± 1	29.9 ± 1	30 ± 1.1	31.5 ± 1	30.6 ± 0.9	30.5 ± 0.8	29.6 ± 0.8	30 ± 0.8	28.6 ± 0.8	29.5 ± 0.9	29.8 ± 1	28.6 ± 1	33.8 ± 1.1	34.1 ± 1.1	P < 0.05
100–199%	20.7 ± 0.7	23.9 ± 0.7	24.4 ± 0.7	24.7 ± 0.7	23.2 ± 0.7	23.6 ± 0.7	23.2 ± 0.7	24 ± 0.9	23.6 ± 0.8	24.3 ± 0.9	26.6 ± 0.8	25.6 ± 0.7	24.1 ± 0.7	23.9 ± 0.7	22.9 ± 0.7	23.4 ± 0.8	24.1 ± 0.8	25.5 ± 0.9	25.2 ± 0.9	26.1 ± 0.9	27.9 ± 0.9	
200–399%	16.9 ± 0.5	19.8 ± 0.5	19.2 ± 0.5	20.2 ± 0.5	20 ± 0.5	20.4 ± 0.6	20.2 ± 0.5	19.9 ± 0.6	20 ± 0.7	19.8 ± 0.6	20.8 ± 0.6	20.4 ± 0.6	18.8 ± 0.5	18.6 ± 0.5	17.7 ± 0.5	17.2 ± 0.6	17.1 ± 0.6	19.2 ± 0.6	18.3 ± 0.6	21.2 ± 0.6	20.8 ± 0.6	
400% or more	13.6 ± 0.4	16.8 ± 0.5	16.4 ± 0.4	16.6 ± 0.5	16.8 ± 0.5	17.5 ± 0.5	16.9 ± 0.5	16.9 ± 0.6	16.1 ± 0.5	16.9 ± 0.5	16.3 ± 0.5	17 ± 0.5	16.1 ± 0.5	14.1 ± 0.5	13.4 ± 0.5	13.5 ± 0.5	14.3 ± 0.5	13.9 ± 0.5	13.6 ± 0.5	16 ± 0.5	16 ± 0.4	
Health insurance status at the time of interview (18–64 years) (one or more ED visits percentage (SE))																						
Insured	16.1 ± 0.3	19.5 ± 0.3	19.2 ± 0.3	19.6 ± 0.3	19.7 ± 0.4	20.1 ± 0.4	20 ± 0.3	19.9 ± 0.4	19.4 ± 0.4	20.3 ± 0.4	20.5 ± 0.4	20.8 ± 0.4	19.4 ± 0.4	19.1 ± 0.4	18.1 ± 0.4	18.2 ± 0.4	18 ± 0.4	18.7 ± 0.4	17.6 ± 0.4	19.9 ± 0.4	20.5 ± 0.4	P < 0.05
Private	14.5 ± 0.3	17.6 ± 0.3	17.2 ± 0.3	17.4 ± 0.3	17.4 ± 0.4	18 ± 0.4	17.3 ± 0.4	17.2 ± 0.4	16.9 ± 0.4	17 ± 0.4	16.7 ± 0.4	17.4 ± 0.4	15.7 ± 0.4	15 ± 0.4	14.1 ± 0.4	14.5 ± 0.4	14.1 ± 0.4	14.5 ± 0.4	14.1 ± 0.4	16.2 ± 0.4	16.5 ± 0.4	
Medicaid	35.4 ± 1.4	42.2 ± 1.5	39.6 ± 1.3	40.4 ± 1.4	39.7 ± 1.3	36.8 ± 1.3	40.1 ± 1.3	39 ± 1.4	37.9 ± 1.4	39.7 ± 1.6	41.5 ± 1.4	40.2 ± 1.2	37.6 ± 1.2	39.7 ± 1.1	38 ± 1.1	34.9 ± 1.1	34.9 ± 1.1	35.4 ± 1.2	32.6 ± 1.2	37 ± 1.2	37.7 ± 1.1	
Uninsured	18.3 ± 0.7	19.3 ± 0.7	18.6 ± 0.7	20.4 ± 0.7	18.1 ± 0.7	19 ± 0.7	19.5 ± 0.7	18.9 ± 0.8	20.3 ± 0.8	19.1 ± 0.8	21.2 ± 0.8	21.3 ± 0.7	21 ± 0.7	18.8 ± 0.7	18.5 ± 0.7	16.5 ± 0.7	18 ± 0.9	17.6 ± 1	19.1 ± 1	20.6 ± 1.1	22.1 ± 1	
Location of residence																						
Within MSA	16.6 ± 0.3	19.6 ± 0.3	19.3 ± 0.3	19.8 ± 0.3	19.5 ± 0.3	20.3 ± 0.3	20.1 ± 0.3	20.1 ± 0.4	19.7 ± 0.4	20.1 ± 0.4	20.9 ± 0.4	20.8 ± 0.3	20 ± 0.3	18.9 ± 0.3	18.3 ± 0.3	18.1 ± 0.3	18.2 ± 0.3	18.9 ± 0.3	18.2 ± 0.4	20.8 ± 0.4	21.3 ± 0.3	P < 0.05
Outside MSA	19.5 ± 0.7	22.5 ± 0.6	21.3 ± 0.7	23.4 ± 0.7	22.3 ± 0.8	22.6 ± 0.8	22.3 ± 0.7	22.6 ± 0.9	22.9 ± 0.8	23.9 ± 1	24 ± 0.7	25.5 ± 0.9	23 ± 0.8	22.9 ± 0.9	22.2 ± 0.9	22 ± 0.8	22.8 ± 0.9	23.4 ± 1	21.5 ± 0.9	25 ± 1	24.8 ± 1	

Overall, while there have been fluctuations in ED visits over the years, the trend shows a slight decrease in visits from 1999 to 2019. Additionally, there are notable differences in visit patterns between males and females and across different age groups.

Based on age

**Figure 1:**
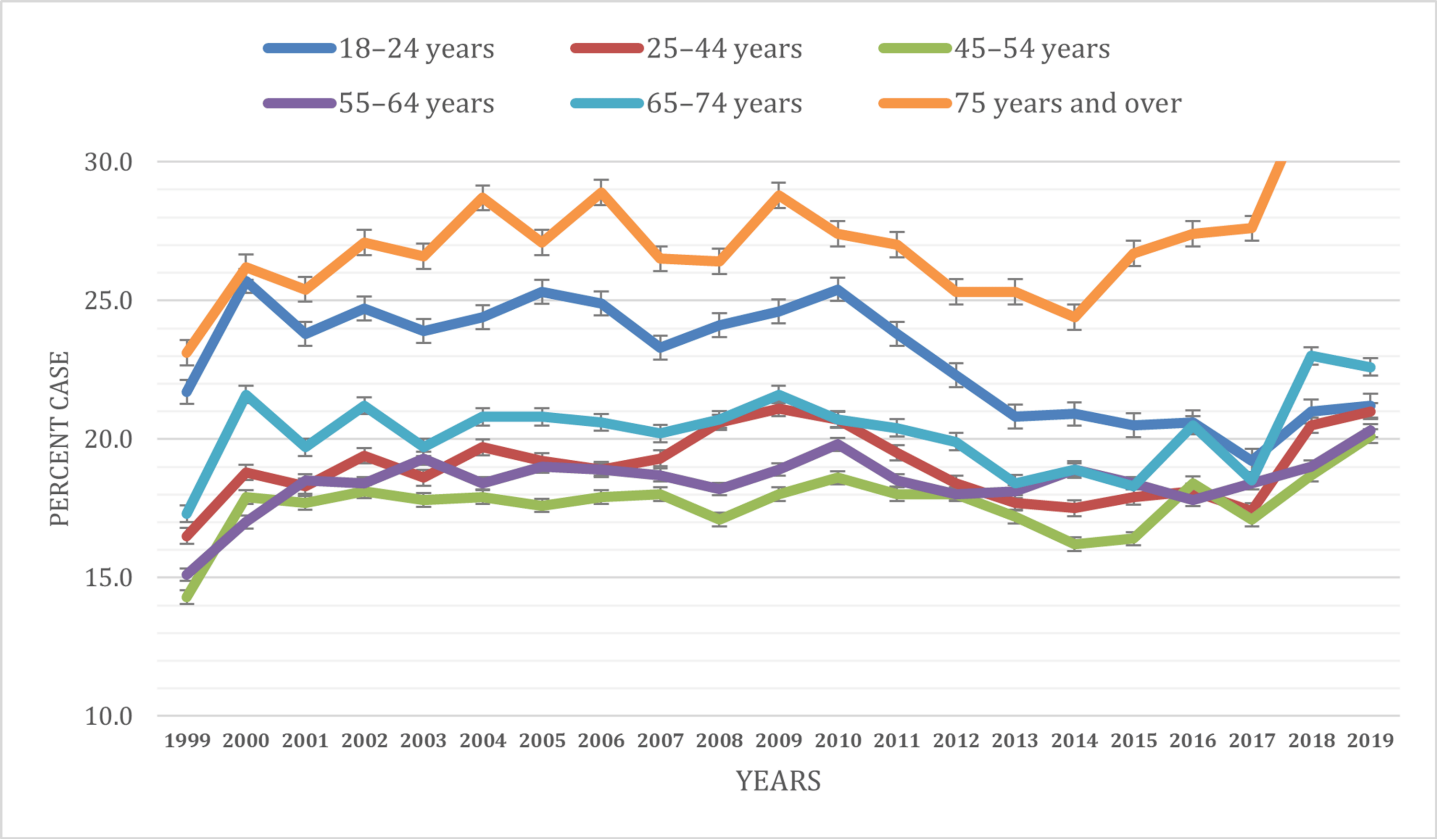
ED visit trends based on age during the study period (1999-2019) ED, emergency department

The prevalence of ED visits varied across different age groups over the study period from 1999 to 2019 (Figure 1). The prevalence of ED visits across different age groups shows distinct patterns over the years. Among individuals aged 18-24 years, the prevalence ranges from 21.7% ± 0.9% to 25.7% ± 0.9%, with a significant increase observed from 1999 to 2000, followed by fluctuations but remaining relatively stable overall. Similarly, for those aged 25-44 years, the prevalence fluctuates between 16.5% ± 0.4% and 21.1% ± 0.6%, with a notable peak in 2010. In the 45-54 years age group, the prevalence ranges from 14.3% ± 0.6% to 20.1% ± 0.7%, displaying consistent patterns over the years. Among individuals aged 55-64 years, the prevalence varies from 15.1% ± 0.6% to 20.3% ± 0.6%, showing fluctuations but maintaining a relatively stable trend. For those aged 65-74 years, the prevalence ranges from 17.3% ± 0.8% to 23.0% ± 0.8%, demonstrating moderate variability over the years. Notably, individuals aged 75 years and over consistently exhibit the highest prevalence, ranging from 24.4% ± 0.9% to 32.5% ± 1.1%. Significant differences in ED visit prevalence were observed across the years within each age group (P < 0.05). These findings underscore age-specific variations in ED utilization and highlight the importance of tailored healthcare strategies for different age cohorts.

Based on race

**Figure 2:**
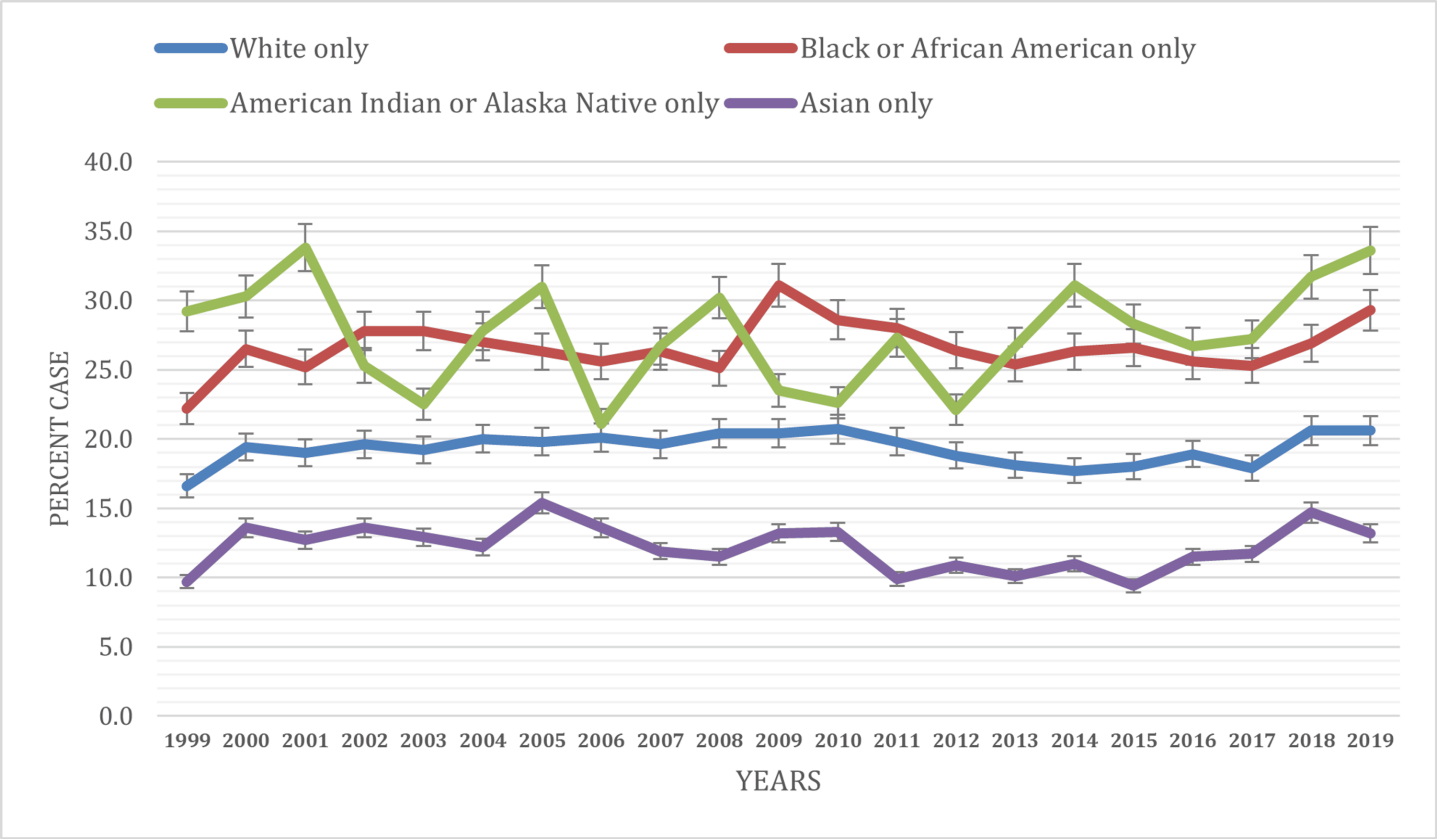
ED visit trends based on race during the study period (1999-2019) ED, emergency department

The prevalence of ED visits varied significantly across different racial groups over the study period from 1999 to 2019 (Figure 2). The prevalence of ED visits varies among different racial and ethnic groups over the years. For individuals identifying as White only, the prevalence ranges from 16.6% ± 0.3% to 20.7% ± 0.4%, with a slight increase observed from 1999 to 2011, followed by fluctuations but remaining relatively stable overall. In contrast, those identifying as Black or African American only exhibit higher prevalence rates, ranging from 22.2% ± 0.8% to 29.3% ± 1%, with a notable peak in 2009. American Indian or Alaska Native individuals show fluctuating prevalence rates, ranging from 21.1% ± 3.7% to 33.6% ± 3.7%, with considerable variability over the years. Asian-only individuals consistently demonstrate lower prevalence rates compared to other groups, fluctuating between 9.4% ± 0.8% and 15.4% ± 1.4%. Significant differences in prevalence were observed across the years within all racial groups (P < 0.05), indicating varying patterns in ED utilization among individuals. These findings underscore the importance of considering racial disparities in ED utilization and the need for targeted interventions to address the unique healthcare needs of different racial groups.

Based on the poverty level

**Figure 3:**
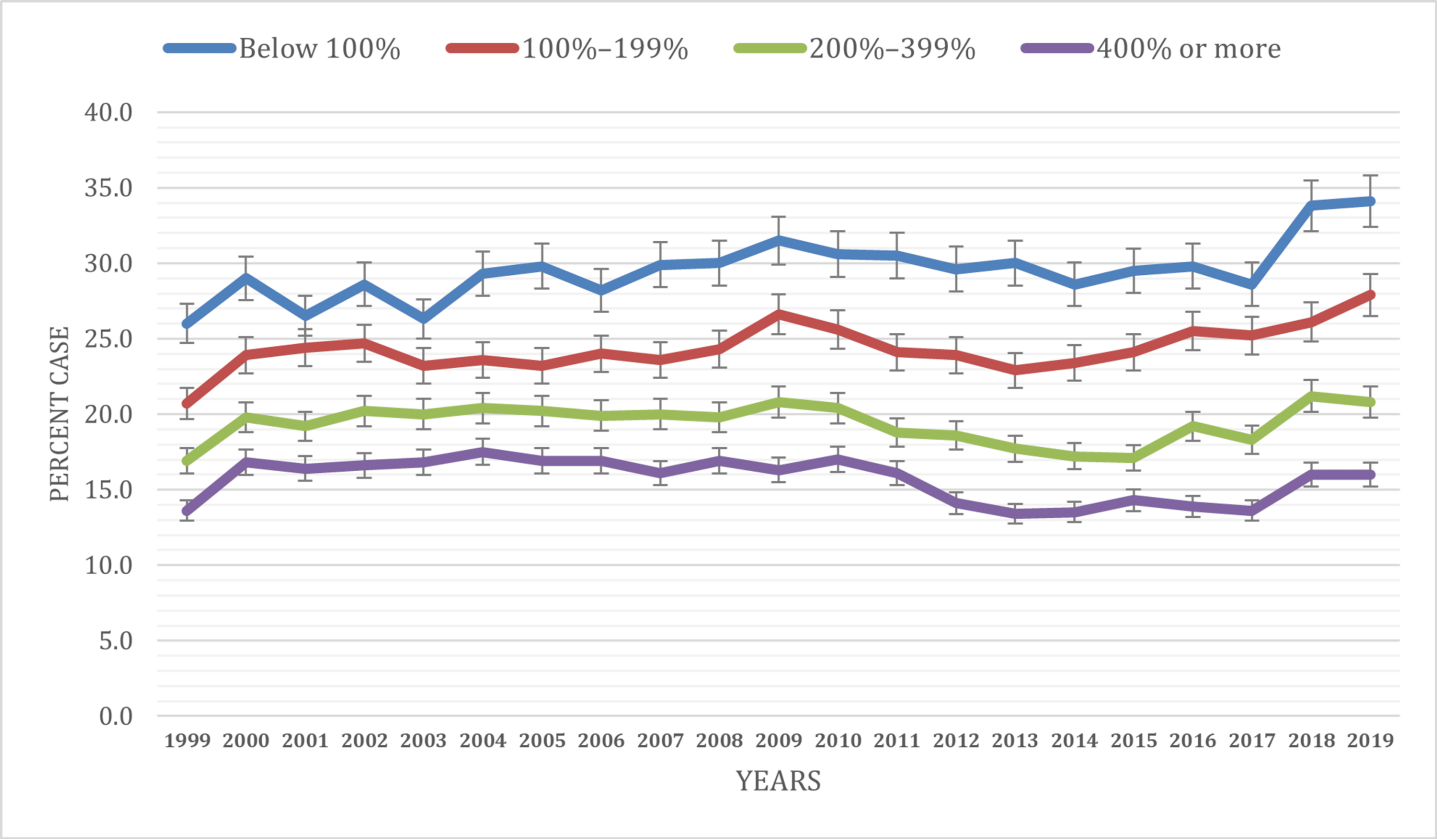
ED visit trends based on poverty during the study period (1999-2019) ED, emergency department

The prevalence of ED visits varies across different levels of poverty over the years (Figure 3). Individuals living below 100% of the poverty level consistently exhibit higher prevalence rates, ranging from 26% ± 0.9% to 34.1% ± 1.1%, with a notable increase observed from 1999 to 2010, followed by fluctuations but remaining relatively elevated. Those living within the range of 100% to 199% of the poverty level also demonstrate notable prevalence rates, ranging from 20.7% ± 0.7% to 27.9% ± 0.9%, with a consistent increase observed from 1999 to 2019. Individuals within the 200% to 399% poverty level range exhibit prevalence rates ranging from 16.9% ± 0.5% to 21.2% ± 0.6%, showing fluctuations but remaining relatively stable overall. Finally, those at 400% or more of the poverty level consistently display lower prevalence rates, ranging from 13.6% ± 0.4% to 17.5% ± 0.5%. Significant differences in prevalence were noted across the years between income categories (P < 0.05), indicating diverse patterns in ED utilization among individuals with different socioeconomic statuses. These findings highlight disparities in ED utilization across different socioeconomic groups, emphasizing the need for targeted healthcare interventions to address the diverse needs of individuals living in poverty.

Based on health insurance status

**Figure 4:**
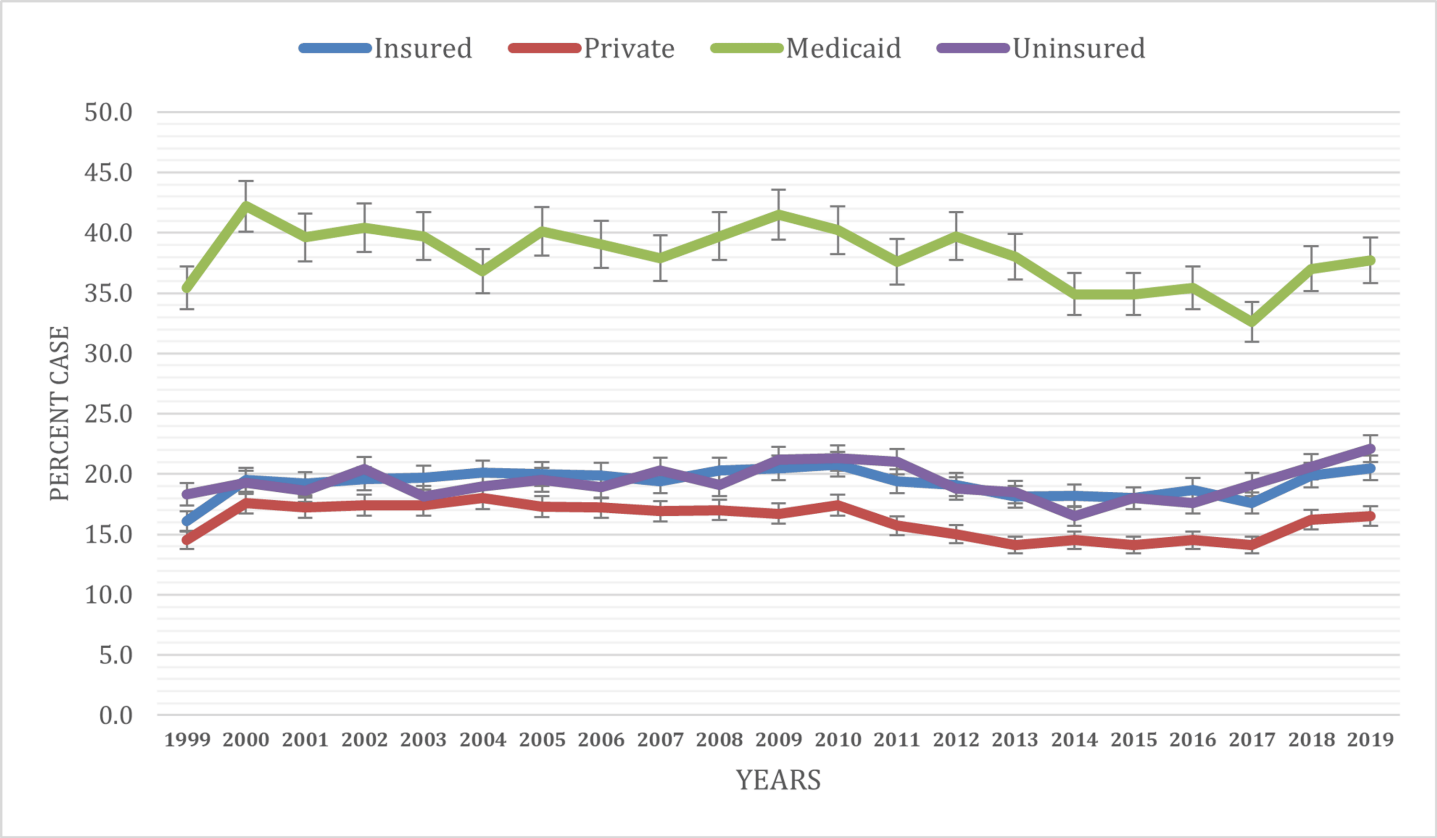
ED visit trends based on insurance during the study period (1999-2019) ED, emergency department

The prevalence of ED visits varies significantly based on insurance status over the years from 1999 to 2019 (Figure 4). Insured individuals consistently exhibit relatively lower prevalence rates, ranging from 16.1% ± 0.3% to 20.5% ± 0.4% throughout the period. Private insurance holders generally demonstrate slightly lower prevalence rates, ranging from 14.5% ± 0.3% to 16.5% ± 0.4%, compared to the insured average. Medicaid beneficiaries consistently exhibit higher prevalence rates, ranging from 35.4% ± 1.4% to 42.2% ± 1.5%, with fluctuations but generally remaining elevated throughout the years. On the other hand, uninsured individuals consistently display higher prevalence rates compared to the insured population, ranging from 18.3% ± 0.7% to 22.1% ± 1%, with fluctuations but remaining relatively elevated. Significant differences in prevalence were noted across the years between insurance categories (P < 0.05). These findings underscore the impact of insurance coverage on healthcare access and utilization, highlighting the importance of ensuring comprehensive insurance coverage to mitigate disparities in ED visits.

Based on the location of residence

Over the two decades from 1999 to 2019, the prevalence of ED visits varied between individuals residing within and outside MSAs. Within MSAs, the prevalence rates ranged from 16.6% ± 0.3% to 21.3% ± 0.3%, with a statistically significant average of 19.5%. Despite fluctuations, there was a noticeable increasing trend in ED visits among individuals within MSAs, particularly evident from the relatively lower rates in 1999 to the higher rates in 2019. Conversely, individuals residing outside MSAs consistently demonstrated higher prevalence rates, ranging from 19.5% ± 0.7% to 24.8% ± 1%. Despite some fluctuations, there was a trend towards increasing prevalence rates over the years, highlighting the persistent healthcare needs of individuals residing in non-MSA areas. Significant differences in prevalence were noted across the years between the locations of residence categories (P < 0.05). These findings emphasize the importance of regional disparities in healthcare utilization and the need for targeted interventions to address the healthcare needs of both urban and rural populations.

## Discussion

This study provides insights into ED visits among adults, compares them to previous studies, and discusses their implications for healthcare policy and practice. Our analysis revealed several noteworthy trends in ED visits among adults over the past two decades. First, we observed a steady increase in the overall rate of ED visits among adults aged 18 and over, consistent with previous studies [10,11]. However, a United States Department of Health and Human Services report on trends in ED utilization revealed stability in the proportion of ED visits across categories from 2009 to 2018. This suggests consistency in the distribution of visits, potentially indicating enduring patterns in healthcare-seeking behavior [4]. This trend suggests a growing reliance on emergency care services in the United States, potentially reflecting challenges in accessing primary care, inadequate preventive care, or worsening health conditions among adults.

Our study revealed variations in ED utilization across demographic and socioeconomic groups. Females exhibited slightly higher ED visit rates, contrasting with mixed evidence from prior studies indicating varied reasons for ED utilization beyond gender disparities. Males showed a prevalence ranging from 16.1 ± 0.4% to 19.4 ± 0.5% between 1999 and 2019. In contrast, females exhibited a higher prevalence, ranging from 18.2 ± 0.4% to 24.0 ± 0.4% during the same period. Potential reasons for the higher ED visit rates among females include limited access to primary care, differences in health conditions, and a higher prevalence of chronic diseases. Additionally, females might experience unique health issues requiring urgent care, such as reproductive health concerns, which can contribute to increased ED utilization. These findings underscore the complexity of factors influencing ED visits and highlight the need for targeted interventions addressing diverse healthcare needs [12-15]. Furthermore, our analysis highlights significant racial disparities in ED visits. White individuals consistently had lower ED visit rates compared to other racial groups; American Indian or Alaska Native and Black or African American individuals consistently exhibited the highest prevalence, followed by Asian individuals [5,16]. Zhang et al.’s study revealed that Asian patients were 1.21 times more inclined than White patients to require hospital admission after an ED visit [17]. These disparities underscore the importance of addressing structural inequities in healthcare access and delivery to ensure equitable emergency care provision for all racial and ethnic groups. Further research is warranted to explore the underlying factors contributing to these disparities and to inform targeted interventions to reduce healthcare inequities.

Distinct age-related ED visit variations were observed. Young adults (18-24 years) showed high visit rates due to risky behavior, limited primary care access, and mental health issues. Conversely, older adults (65 years and above) had significant ED utilization due to chronic conditions and complex healthcare needs. Comparisons with earlier studies reveal persistent age-specific ED utilization patterns [18-20]. Aligned with our results, Alnahari and A'aqoulah’s study indicated that individuals aged 60 and above are less prone to prolonged stays in the ED than those aged 29 or younger [21]. Socioeconomic status emerged as a significant determinant of ED utilization. Consistent with previous research, we found that individuals with lower socioeconomic status, as indicated by poverty level, exhibited higher ED visit rates than those with higher income levels [5,22]. This disparity underscores the critical role of socioeconomic factors in shaping healthcare access and utilization patterns. Moreover, our analysis revealed variations in ED utilization based on health insurance coverage. Medicaid recipients consistently exhibited higher ED visit rates compared to other insurance categories, a finding consistent with existing literature [5,22,23]. Hsiang et al.’s meta-analysis found that Medicaid patients face more challenges scheduling appointments than privately insured individuals across various medical situations [24]. This disparity may stem from various factors, including limited access to primary care providers accepting Medicaid, complex health needs among Medicaid beneficiaries, and socioeconomic challenges affecting healthcare-seeking behaviors.

Our study highlighted geographic disparities in ED utilization, with individuals residing outside MSAs showing higher ED visit rates than those within MSAs. Limited access to healthcare facilities, transportation barriers, and shortages of primary care providers in rural areas may contribute to the higher reliance on ED services among rural residents. These trends emphasize the need for targeted interventions to enhance healthcare access, promote preventive care, and reduce unnecessary ED utilization. Policymakers should prioritize addressing barriers to primary care access, expanding healthcare infrastructure, and incentivizing primary care providers to accept Medicaid. Additionally, implementing telehealth initiatives can improve access, particularly in rural areas. Improving care coordination and transitions from ED to primary care settings can help alleviate ED overcrowding. Strengthening community-based interventions, such as health education and chronic disease management programs, can reduce preventable ED visits and enhance health outcomes. By comparing our findings to prior research, we underscore the importance of ongoing monitoring and tailored interventions to address disparities and optimize healthcare delivery.

Limitations

The study has some limitations. First, its retrospective design relies on the accuracy and completeness of historical data, which may affect the reliability of the findings. Second, while the study identifies associations between factors and ED visit rates, it does not establish causality or explore the underlying reasons for these disparities. Third, the reliance on data from the NCHS database may introduce biases due to variations in data collection and reporting practices, potentially impacting the study’s generalizability. Fourth, the study does not account for other relevant variables, such as specific health conditions or the availability of primary care services, which could influence ED utilization patterns. Finally, the temporal scope of the study, covering 1999 to 2019, excludes more recent data and may miss emerging trends. There is also the possibility of underreporting or overreporting ED visits due to factors like social desirability bias or recall bias. These limitations suggest caution in interpreting the findings and indicate areas for future research.

## Conclusions

Our comprehensive analysis of ED visits among adults, utilizing the NCHS database, revealed several significant findings. We observed a steady increase in overall ED utilization over two decades, with notable variations across demographic, socioeconomic, and geographic groups. Age-specific patterns highlighted disparities in ED visits, underscoring the importance of tailored healthcare interventions. Moreover, gender and racial disparities in ED utilization emphasize the need for equitable access to healthcare services. These findings provide valuable insights for policymakers, healthcare providers, and stakeholders to develop targeted strategies aimed at optimizing healthcare delivery, improving access to primary care, and reducing unnecessary ED visits among adults. Future research should delve into the underlying factors contributing to trends and patterns in ED visits. By examining socioeconomic, demographic, and health-related determinants, researchers can identify key disparities and develop targeted interventions. Additionally, longitudinal studies could provide insights into how changes over time impact ED utilization, ultimately guiding policy and improving healthcare delivery systems.
